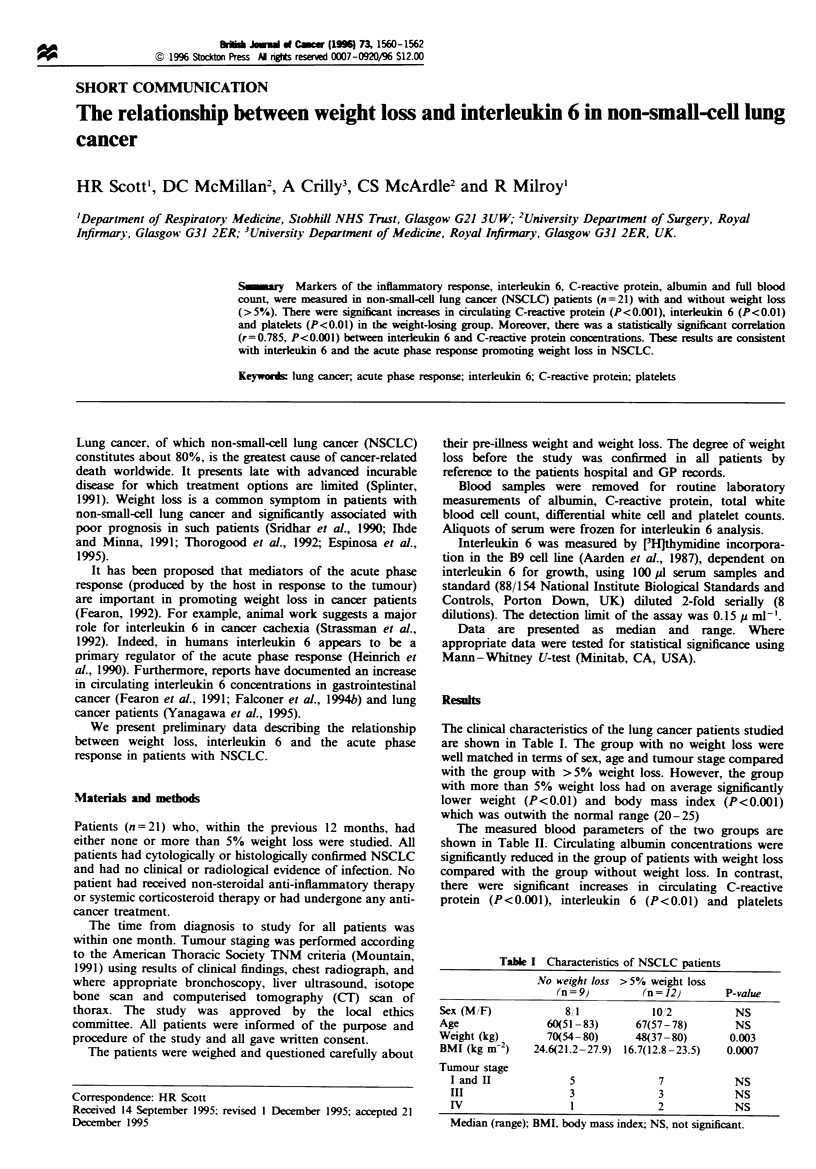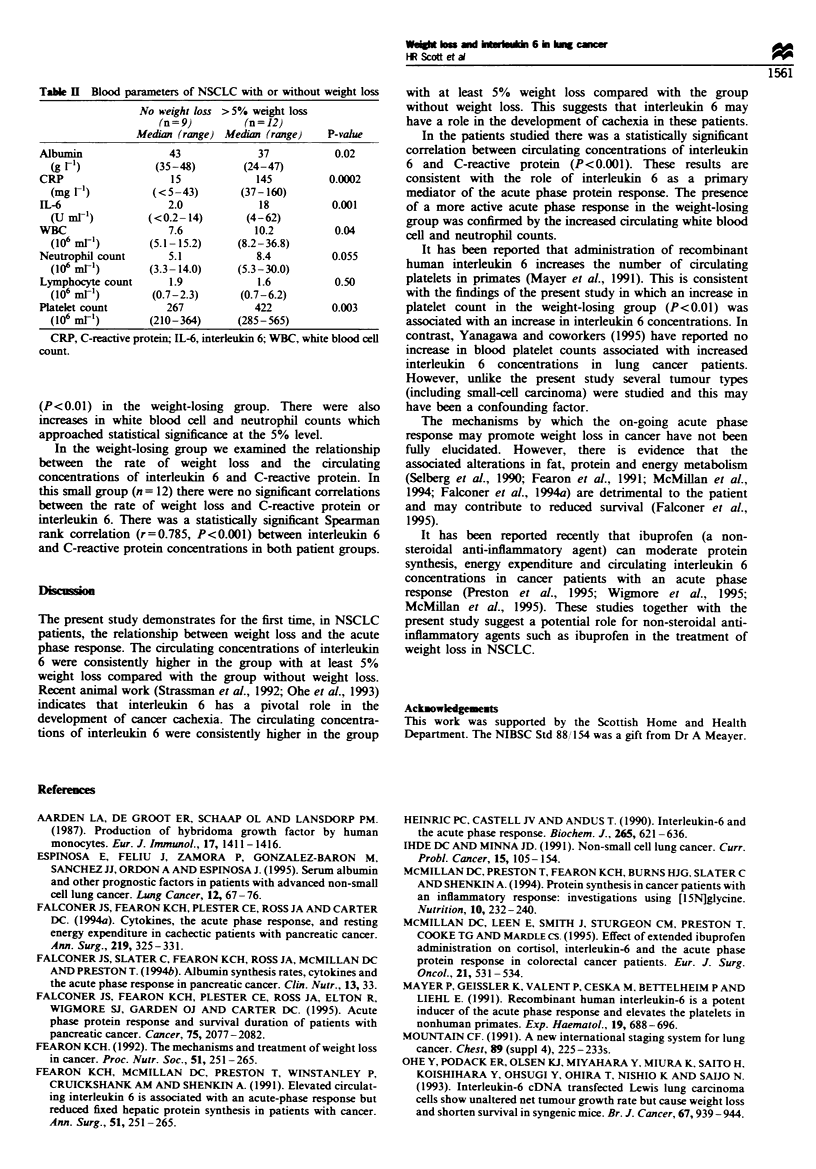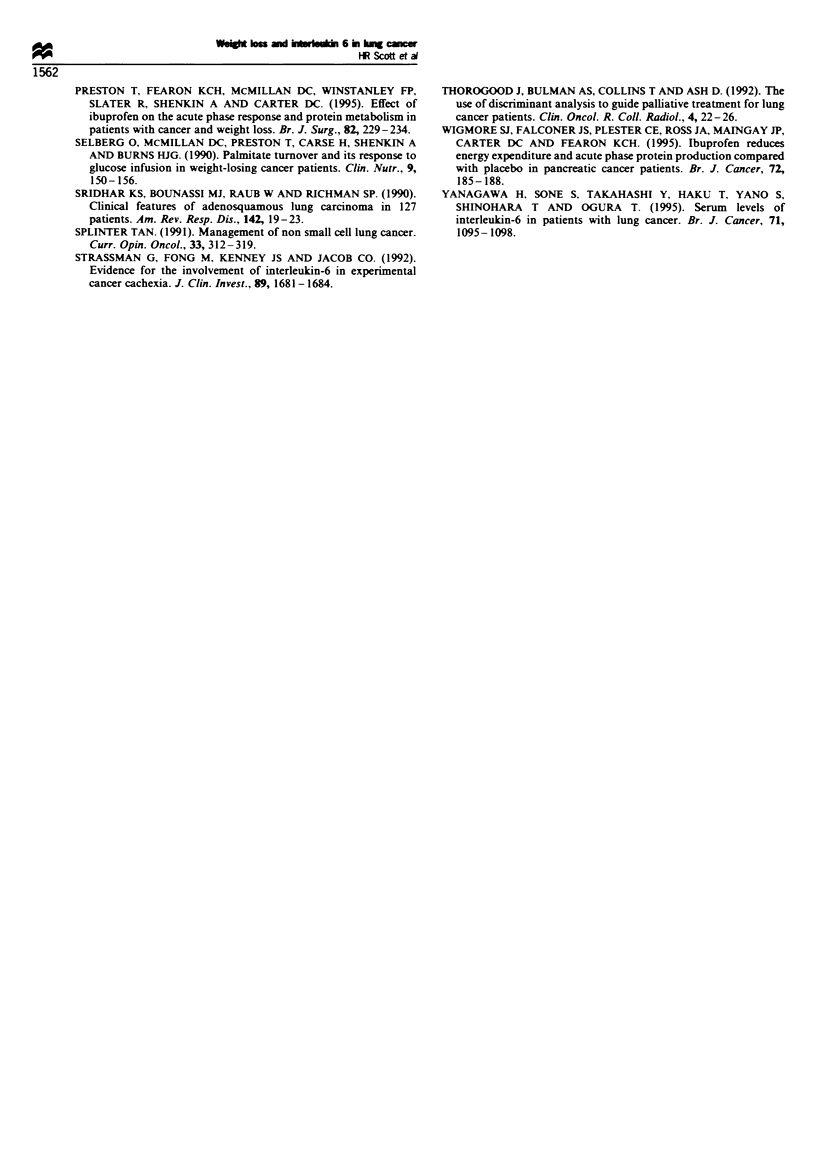# The relationship between weight loss and interleukin 6 in non-small-cell lung cancer.

**DOI:** 10.1038/bjc.1996.294

**Published:** 1996-06

**Authors:** H. R. Scott, D. C. McMillan, A. Crilly, C. S. McArdle, R. Milroy

**Affiliations:** Department of Respiratory Medicine, Stobhill NHS Trust, Glasgow, UK.

## Abstract

Markers of the inflammatory response, interleukin 6, C-reactive protein, albumin and full blood count, were measured in non-small-cell lung cancer (NSCLC) patients (n = 21) with and without weight loss ( > 5%). There were significant increases in circulating C-reactive protein (P < 0.001), interleukin 6 (P < 0.01) and platelets (P < 0.01) in the weight-losing group. Moreover, there was a statistically significant correlation (r = 0.785, P < 0.001) between interleukin 6 and C-reactive protein concentrations. These results are consistent with interleukin 6 and the acute phase response promoting weight loss in NSCLC.


© 1996 Stockton Press All rights reserved 0007-0920/96 $12.00

SHORT COMMUNICATION

### The relationship between weight loss and interleukin 6 in non-small-cell lung cancer

HR Scott[1], DC McMillan[2], A Crilly[3], CS McArdle[2] and R Milroy[1]

[1]*Department of Respiratory Medicine, Stobhill NHS Trust, Glasgow G21 3UW;* [2]*University Department of Surgery, Royal Infirmary, Glasgow G31 2ER;* [3]*University Department of Medicine, Royal Infirmary, Glasgow G31 2ER, UK.*

**Summary** Markers of the inflammatory response, interleukin 6, C-reactive protein, albumin and full blood count, were measured in non-small-cell lung cancer (NSCLC) patients ($n=21$) with and without weight loss (>5%). There were significant increases in circulating C-reactive protein ($P<0.001$), interleukin 6 ($P<0.01$) and platelets ($P<0.01$) in the weight-losing group. Moreover, there was a statistically significant correlation ($r=0.785$, $P<0.001$) between interleukin 6 and C-reactive protein concentrations. These results are consistent with interleukin 6 and the acute phase response promoting weight loss in NSCLC.

**Keywords:** lung cancer; acute phase response; interleukin 6; C-reactive protein; platelets

Lung cancer, of which non-small-cell lung cancer (NSCLC) constitutes about 80%, is the greatest cause of cancer-related death worldwide. It presents late with advanced incurable disease for which treatment options are limited (Splinter, 1991). Weight loss is a common symptom in patients with non-small-cell lung cancer and significantly associated with poor prognosis in such patients (Sridhar *et al.*, 1990; Ihde and Minna, 1991; Thorogood *et al.*, 1992; Espinosa *et al.*, 1995).

It has been proposed that mediators of the acute phase response (produced by the host in response to the tumour) are important in promoting weight loss in cancer patients (Fearon, 1992). For example, animal work suggests a major role for interleukin 6 in cancer cachexia (Strassman *et al.*, 1992). Indeed, in humans interleukin 6 appears to be a primary regulator of the acute phase response (Heinrich *et al.*, 1990). Furthermore, reports have documented an increase in circulating interleukin 6 concentrations in gastrointestinal cancer (Fearon *et al.*, 1991; Falconer *et al.*, 1994*b*) and lung cancer patients (Yanagawa *et al.*, 1995).

We present preliminary data describing the relationship between weight loss, interleukin 6 and the acute phase response in patients with NSCLC.

### Materials and methods

Patients ($n=21$) who, within the previous 12 months, had either none or more than 5% weight loss were studied. All patients had cytologically or histologically confirmed NSCLC and had no clinical or radiological evidence of infection. No patient had received non-steroidal anti-inflammatory therapy or systemic corticosteroid therapy or had undergone any anti-cancer treatment.

The time from diagnosis to study for all patients was within one month. Tumour staging was performed according to the American Thoracic Society TNM criteria (Mountain, 1991) using results of clinical findings, chest radiograph, and where appropriate bronchoscopy, liver ultrasound, isotope bone scan and computerised tomography (CT) scan of thorax. The study was approved by the local ethics committee. All patients were informed of the purpose and procedure of the study and all gave written consent.

The patients were weighed and questioned carefully about their pre-illness weight and weight loss. The degree of weight loss before the study was confirmed in all patients by reference to the patients hospital and GP records.

Blood samples were removed for routine laboratory measurements of albumin, C-reactive protein, total white blood cell count, differential white cell and platelet counts. Aliquots of serum were frozen for interleukin 6 analysis.

Interleukin 6 was measured by [$^3$H]thymidine incorporation in the B9 cell line (Aarden *et al.*, 1987), dependent on interleukin 6 for growth, using 100 $\mu$l serum samples and standard (88/154 National Institute Biological Standards and Controls, Porton Down, UK) diluted 2-fold serially (8 dilutions). The detection limit of the assay was 0.15 $\mu$ ml$^{-1}$.

Data are presented as median and range. Where appropriate data were tested for statistical significance using Mann-Whitney *U*-test (Minitab, CA, USA).

### Results

The clinical characteristics of the lung cancer patients studied are shown in Table I. The group with no weight loss were well matched in terms of sex, age and tumour stage compared with the group with >5% weight loss. However, the group with more than 5% weight loss had on average significantly lower weight ($P<0.01$) and body mass index ($P<0.001$) which was outwith the normal range (20-25)

The measured blood parameters of the two groups are shown in Table II. Circulating albumin concentrations were significantly reduced in the group of patients with weight loss compared with the group without weight loss. In contrast, there were significant increases in circulating C-reactive protein ($P<0.001$), interleukin 6 ($P<0.01$) and platelets

**Table I** Characteristics of NSCLC patients

| | *No weight loss* (n=*9*) | >5% weight loss (n=*12*) | P-*value* |
|---|---|---|---|
| Sex (M/F) | 8/1 | 10/2 | NS |
| Age | 60(51-83) | 67(57-78) | NS |
| Weight (kg) | 70(54-80) | 48(37-80) | 0.003 |
| BMI (kg m$^{-2}$) | 24.6(21.2-27.9) | 16.7(12.8-23.5) | 0.0007 |
| Tumour stage | | | |
| I and II | 5 | 7 | NS |
| III | 3 | 3 | NS |
| IV | 1 | 2 | NS |

Median (range); BMI, body mass index; NS, not significant.

Correspondence: HR Scott
Received 14 September 1995; revised 1 December 1995; accepted 21 December 1995

**Table II** Blood parameters of NSCLC with or without weight loss

| | *No weight loss* (n=9) *Median (range)* | >5% weight loss (n=12) *Median (range)* | P-*value* |
|---|---|---|---|
| Albumin | 43 | 37 | 0.02 |
| (g $l^{-1}$) | (35–48) | (24–47) | |
| CRP | 15 | 145 | 0.0002 |
| (mg $l^{-1}$) | (<5–43) | (37–160) | |
| IL-6 | 2.0 | 18 | 0.001 |
| (U $ml^{-1}$) | (<0.2–14) | (4–62) | |
| WBC | 7.6 | 10.2 | 0.04 |
| ($10^6$ $ml^{-1}$) | (5.1–15.2) | (8.2–36.8) | |
| Neutrophil count | 5.1 | 8.4 | 0.055 |
| ($10^6$ $ml^{-1}$) | (3.3–14.0) | (5.3–30.0) | |
| Lymphocyte count | 1.9 | 1.6 | 0.50 |
| ($10^6$ $ml^{-1}$) | (0.7–2.3) | (0.7–6.2) | |
| Platelet count | 267 | 422 | 0.003 |
| ($10^6$ $ml^{-1}$) | (210–364) | (285–565) | |

CRP, C-reactive protein; IL-6, interleukin 6; WBC, white blood cell count.

($P<0.01$) in the weight-losing group. There were also increases in white blood cell and neutrophil counts which approached statistical significance at the 5% level.

In the weight-losing group we examined the relationship between the rate of weight loss and the circulating concentrations of interleukin 6 and C-reactive protein. In this small group ($n=12$) there were no significant correlations between the rate of weight loss and C-reactive protein or interleukin 6. There was a statistically significant Spearman rank correlation ($r=0.785$, $P<0.001$) between interleukin 6 and C-reactive protein concentrations in both patient groups.

### Discussion

The present study demonstrates for the first time, in NSCLC patients, the relationship between weight loss and the acute phase response. The circulating concentrations of interleukin 6 were consistently higher in the group with at least 5% weight loss compared with the group without weight loss. Recent animal work (Strassman *et al.*, 1992; Ohe *et al.*, 1993) indicates that interleukin 6 has a pivotal role in the development of cancer cachexia. The circulating concentrations of interleukin 6 were consistently higher in the group with at least 5% weight loss compared with the group without weight loss. This suggests that interleukin 6 may have a role in the development of cachexia in these patients.

In the patients studied there was a statistically significant correlation between circulating concentrations of interleukin 6 and C-reactive protein ($P<0.001$). These results are consistent with the role of interleukin 6 as a primary mediator of the acute phase protein response. The presence of a more active acute phase response in the weight-losing group was confirmed by the increased circulating white blood cell and neutrophil counts.

It has been reported that administration of recombinant human interleukin 6 increases the number of circulating platelets in primates (Mayer *et al.*, 1991). This is consistent with the findings of the present study in which an increase in platelet count in the weight-losing group ($P<0.01$) was associated with an increase in interleukin 6 concentrations. In contrast, Yanagawa and coworkers (1995) have reported no increase in blood platelet counts associated with increased interleukin 6 concentrations in lung cancer patients. However, unlike the present study several tumour types (including small-cell carcinoma) were studied and this may have been a confounding factor.

The mechanisms by which the on-going acute phase response may promote weight loss in cancer have not been fully elucidated. However, there is evidence that the associated alterations in fat, protein and energy metabolism (Selberg *et al.*, 1990; Fearon *et al.*, 1991; McMillan *et al.*, 1994; Falconer *et al.*, 1994*a*) are detrimental to the patient and may contribute to reduced survival (Falconer *et al.*, 1995).

It has been reported recently that ibuprofen (a non-steroidal anti-inflammatory agent) can moderate protein synthesis, energy expenditure and circulating interleukin 6 concentrations in cancer patients with an acute phase response (Preston *et al.*, 1995; Wigmore *et al.*, 1995; McMillan *et al.*, 1995). These studies together with the present study suggest a potential role for non-steroidal anti-inflammatory agents such as ibuprofen in the treatment of weight loss in NSCLC.

**Acknowledgements**
This work was supported by the Scottish Home and Health Department. The NIBSC Std 88/154 was a gift from Dr A Meayer.

### References

AARDEN LA, DE GROOT ER, SCHAAP OL AND LANSDORP PM. (1987). Production of hybridoma growth factor by human monocytes. *Eur. J. Immunol.*, **17**, 1411–1416.

ESPINOSA E, FELIU J, ZAMORA P, GONZALEZ-BARON M, SANCHEZ JJ, ORDON A AND ESPINOSA J. (1995). Serum albumin and other prognostic factors in patients with advanced non-small cell lung cancer. *Lung Cancer*, **12**, 67–76.

FALCONER JS, FEARON KCH, PLESTER CE, ROSS JA AND CARTER DC. (1994*a*). Cytokines, the acute phase response, and resting energy expenditure in cachectic patients with pancreatic cancer. *Ann. Surg.*, **219**, 325–331.

FALCONER JS, SLATER C, FEARON KCH, ROSS JA, MCMILLAN DC AND PRESTON T. (1994*b*). Albumin synthesis rates, cytokines and the acute phase response in pancreatic cancer. *Clin. Nutr.*, **13**, 33.

FALCONER JS, FEARON KCH, PLESTER CE, ROSS JA, ELTON R, WIGMORE SJ, GARDEN OJ AND CARTER DC. (1995). Acute phase protein response and survival duration of patients with pancreatic cancer. *Cancer*, **75**, 2077–2082.

FEARON KCH. (1992). The mechanisms and treatment of weight loss in cancer. *Proc. Nutr. Soc.*, **51**, 251–265.

FEARON KCH, MCMILLAN DC, PRESTON T, WINSTANLEY P, CRUICKSHANK AM AND SHENKIN A. (1991). Elevated circulating interleukin 6 is associated with an acute-phase response but reduced fixed hepatic protein synthesis in patients with cancer. *Ann. Surg.*, **51**, 251–265.

HEINRIC PC, CASTELL JV AND ANDUS T. (1990). Interleukin-6 and the acute phase response. *Biochem. J.*, **265**, 621–636.

IHDE DC AND MINNA JD. (1991). Non-small cell lung cancer. *Curr. Probl. Cancer*, **15**, 105–154.

MCMILLAN DC, PRESTON T, FEARON KCH, BURNS HJG, SLATER C AND SHENKIN A. (1994). Protein synthesis in cancer patients with an inflammatory response: investigations using [15N]glycine. *Nutrition*, **10**, 232–240.

MCMILLAN DC, LEEN E, SMITH J, STURGEON CM, PRESTON T, COOKE TG AND MARDLE CS. (1995). Effect of extended ibuprofen administration on cortisol, interleukin-6 and the acute phase protein response in colorectal cancer patients. *Eur. J. Surg. Oncol.*, **21**, 531–534.

MAYER P, GEISSLER K, VALENT P, CESKA M, BETTELHEIM P AND LIEHL E. (1991). Recombinant human interleukin-6 is a potent inducer of the acute phase response and elevates the platelets in nonhuman primates. *Exp. Haematol.*, **19**, 688–696.

MOUNTAIN CF. (1991). A new international staging system for lung cancer. *Chest*, **89** (suppl 4), 225–233s.

OHE Y, PODACK ER, OLSEN KJ, MIYAHARA Y, MIURA K, SAITO H, KOISHIHARA Y, OHSUGI Y, OHIRA T, NISHIO K AND SAIJO N. (1993). Interleukin-6 cDNA transfected Lewis lung carcinoma cells show unaltered net tumour growth rate but cause weight loss and shorten survival in syngenic mice. *Br. J. Cancer*, **67**, 939–944.

PRESTON T, FEARON KCH, MCMILLAN DC, WINSTANLEY FP, SLATER R, SHENKIN A AND CARTER DC. (1995). Effect of ibuprofen on the acute phase response and protein metabolism in patients with cancer and weight loss. *Br. J. Surg.*, **82**, 229–234.

SELBERG O, MCMILLAN DC, PRESTON T, CARSE H, SHENKIN A AND BURNS HJG. (1990). Palmitate turnover and its response to glucose infusion in weight-losing cancer patients. *Clin. Nutr.*, **9**, 150–156.

SRIDHAR KS, BOUNASSI MJ, RAUB W AND RICHMAN SP. (1990). Clinical features of adenosquamous lung carcinoma in 127 patients. *Am. Rev. Resp. Dis.*, **142**, 19–23.

SPLINTER TAN. (1991). Management of non small cell lung cancer. *Curr. Opin. Oncol.*, **33**, 312–319.

STRASSMAN G, FONG M, KENNEY JS AND JACOB CO. (1992). Evidence for the involvement of interleukin-6 in experimental cancer cachexia. *J. Clin. Invest.*, **89**, 1681–1684.

THOROGOOD J, BULMAN AS, COLLINS T AND ASH D. (1992). The use of discriminant analysis to guide palliative treatment for lung cancer patients. *Clin. Oncol. R. Coll. Radiol.*, **4**, 22–26.

WIGMORE SJ, FALCONER JS, PLESTER CE, ROSS JA, MAINGAY JP, CARTER DC AND FEARON KCH. (1995). Ibuprofen reduces energy expenditure and acute phase protein production compared with placebo in pancreatic cancer patients. *Br. J. Cancer*, **72**, 185–188.

YANAGAWA H, SONE S, TAKAHASHI Y, HAKU T, YANO S, SHINOHARA T AND OGURA T. (1995). Serum levels of interleukin-6 in patients with lung cancer. *Br. J. Cancer*, **71**, 1095–1098.